# Determinants of Engagement in Face-to-Face and Online Patient Support Groups

**DOI:** 10.2196/jmir.1718

**Published:** 2011-12-07

**Authors:** Cornelia F Van Uden-Kraan, Constance HC Drossaert, Erik Taal, Willem M Smit, Hein J Bernelot Moens, Mart AFJ Van de Laar

**Affiliations:** ^1^Department of Otolaryngology / Head and Neck SurgeryVU University Medical CentreAmsterdamNetherlands; ^2^Institute for Behavioral ResearchDepartment of Psychology, Health, TechnologyUniversity of TwenteEnschedeNetherlands; ^3^Department of Internal MedicineMedisch Spectrum TwenteEnschedeNetherlands; ^4^Department of RheumalotogyZiekenhuisgroep TwenteHengeloNetherlands; ^5^Department of RheumatologyMedisch Spectrum TwenteEnschedeNetherlands

**Keywords:** patients, support groups, online communities, breast cancer, fibromyalgia, rheumatoid arthritis, theory of planned behavior

## Abstract

**Background:**

Although peer-to-peer contact might empower patients in various ways, studies show that only a few patients actually engage in support groups.

**Objective:**

The objective of our study was to explore factors that facilitate or impede engagement in face-to-face and online peer support, using the Theory of Planned Behavior.

**Methods:**

A questionnaire was completed by 679 patients being treated for arthritis, breast cancer, or fibromyalgia at two Dutch regional hospitals.

**Results:**

Our results showed that only a minority of the patients engaged in organized forms of peer support. In total 10% (65/679) of the respondents had engaged in face-to-face meetings for patients in the past year. Only 4% (30/679) of the respondents had contact with peers via the Internet in the past year. Patients were more positive about face-to-face peer support than about online peer support (*P* < .001). In accordance with the Theory of Planned Behavior, having a more positive attitude (*P* < .01) and feeling more supported by people in the social environment (*P* < .001) increased the intention to participate in both kinds of peer support. In addition, perceived behavioral control (*P* = .01) influenced the intention to participate in online peer support. Nevertheless, the intention to engage in face-to-face and online peer support was only modestly predicted by the Theory of Planned Behavior variables (*R* ^2^ = .33 for face-to-face contact and *R* ^2^ = .26 for online contact).

**Conclusion:**

Although Health 2.0 Internet technology has significantly increased opportunities for having contact with fellow patients, only a minority seem to be interested in organized forms of peer contact (either online or face-to-face). Patients seem somewhat more positive about face-to-face contact than about online contact.

## Introduction

Over the past decades, several studies have shown that patients tend to profit from engagement in peer support groups. Such group participation can offer emotional support, confidence, and strength [1]; can foster hope [2]; and can lead to improved coping [3], less distress [4], and an improved quality of life for the participant [5]. Despite these empowering outcomes of engagement in peer support, studies have shown that many face-to-face peer support groups have only small numbers of participants [6,7].

People who engage in face-to-face peer support groups are more likely than nonparticipants to be female, younger, more highly educated, and of a higher economic status [8-10]. Contradictory findings appeared concerning social support: some studies found that those who participated in support groups experienced less social support in their social environment than nonparticipants [8-10], while others found no differences [6,11,12]. In general, participants seemed to be more anxious about their illness and had greater emotional problems than did nonparticipants [9,13].

With the availability of the Internet, so too the opportunity to share concerns and experiences with peers online has become available. The outcomes of participation in online support groups are in line with the outcomes of participation in face-to-face support groups [14,15]. Engagement in peer support was expected to increase with the emergence of online support groups, as these kinds of groups have specific advantages, such as the absence of geographical barriers, 24-hour availability, and anonymity [16,17]. Yet studies have shown that the use of online patient support groups is limited as well. For example, Atkinson et al [18] found that only 3.8% of their sample of Internet users had ever used an online patient support group, and van de Poll-Franse and van Eenbergen [19] found that only 6% of their sample of cancer patients had actually participated in an online peer support group.

Little is known about determinants of (non)participation in online support groups. We are aware of only two studies that provided some insight. Dutta and Feng [20] showed that participants are younger than nonparticipants. Atkinson et al [18] found that having a poorer health status and a lower income significantly increased use of online support groups for people with similar health or medical issues, while having access to the Internet both at home and at work significantly decreased the use.

In the present study we focused on determinants of engagement in online as well as face-to-face peer support. It is important to gain more insight into the factors that impede or facilitate engagement in peer support, because the numbers of patients that benefit from it might be increased when misconceptions of and barriers to peer support are removed.

As the theoretical basis for the present study, we chose the Theory of Planned Behavior [21]. According to the Theory of Planned Behavior, intention to engage in peer support is determined by three considerations: (1) attitude (ie, thoughts and feelings regarding engagement in peer support), (2) subjective norm (ie, patients’ perceptions about whether significant others would like them to engage in peer support), and (3) perceived behavioral control (ie, the extent to which patients think that they are able to engage in peer support).

The purpose of this quantitative study was (1) to explore differences in intentions, attitudes, social norms, and perceived behavioral control regarding face-to-face and online support groups, and (2) to examine which factors of Theory of Planned Behavior variables, demographic variables, health-related quality of life, and social support predict patients’ intention to engage in both types of peer support. In this respect we were interested not only in which type of peer support could be better explained, but also in whether face-to-face and online peer support would have the same or differing predictors.

## Methods

### Sample and Procedure

Our study was focused on patients with breast cancer, fibromyalgia, or rheumatoid arthritis. We randomly selected 400 patients from each patient group from the electronic database of two regional hospitals. Inclusion criteria were being younger than 75 years and having sufficient knowledge of the Dutch language to be able to fill out the questionnaire. Attending physicians (n = 22) were asked to exclude those patients who did not meet the inclusion criteria and those who for other reasons were deemed unsuitable for participation in our study. Reasons mentioned for exclusion by the physicians were as follows: deceased, aggravation of the illness, comorbidity, mental health problems, wrong diagnosis, or family circumstances. Of the 22 physicians, 2 did not respond, which meant that 30 breast cancer patients were not approached. After exclusion of in total 187 patients we were left with a group of 1013 patients. The attending physicians invited the patients by mail and enclosed the questionnaire. If necessary, this was followed by one reminder. Of the 1013 patients approached, 28 were ineligible because they were deceased or had no valid address. The overall total response rate was 68.9% (n = 679). Of these respondents, 272/350 patients had a diagnosis of rheumatoid arthritis (response rate: 77.7%), 212/333 of fibromyalgia (response rate: 63.7%), and 195/302 of breast cancer (response rate: 64.6%).

All patients were asked for their consent to check the actual date of diagnosis in their medical records. According to the Dutch law for medical research with humans (Wet Medisch-wetenschappelijk onderzoek met Mensen), approval by an ethics committee was not necessary for this survey study.

### Instrument

#### Demographic and Health Characteristics

The respondents were asked to provide information about the demographic characteristics sex, age, marital status, education, and employment. Health-related quality of life was assessed with the SF-12v2. Standardized scores were calculated for the physical and mental well-being varying from 0 (poor) to 100 (excellent), with a mean of 50 and a standard deviation of 10 in the general population of the United States [22].

#### Social Support Factors

Social support factors were measured by the Social Support List-Interaction [23], consisting of 12 items. An example of a social support item is “Does it ever happen that someone shows interest in you?” Respondents could answer on a 4-point scale that ranged from “seldom to never” (score of 1) to “often” (4). The internal consistency (Cronbach alpha) for this construct was alpha = .93. A mean total score was calculated.

#### Use of Peer Support

Respondents were asked to indicate whether they had contact with peers at patient meetings, via the Internet, or at patient organization venues, or whether they had contact with an acquainted peer during the past year and, if so, how frequent this contact was. Respondents could answer on a 4-point scale that ranged from “never” (1) to “regularly” (4).

#### Theory of Planned Behavior Variables

Theory of Planned Behavior variables were measured regarding both face-to-face and online peer contact. Items were derived from the literature (eg, [9,13,14, 24]). For each construct the internal consistency (Cronbach alpha) was determined and a mean total score was calculated.

We asked about patients’ *intention* to have contact with peers face-to-face and via the Internet during the coming year on a 5-point scale that ranged from “certainly no” (1) to “certainly yes” (5). *Attitude* toward face-to-face and online peer support was measured directly with two single items: “Face-to-face contact with peers is valuable” and “Contact with peers via the Internet is valuable.” Attitude was also measured indirectly by assessing advantages and disadvantages. In total, 28 items were formulated (see table 4). Respondents could answer on a 5-point scale that ranged from “totally disagree” (1) to “totally agree” (5). *Advantages of face-to-face peer support* was measured with 9 items (alpha = .93). *Disadvantages of face-to-face peer support* was measured with 5 items (alpha = .74). *Advantages of online peer support* was measured with 9 items (alpha = .92). *Disadvantages of online peer support* was measured with 5 items (alpha = .65).

*Subjective norm* was measured with two items: “People who are important to me think that I certainly should be in contact with peers face-to-face” and “People who are important to me think that I certainly should be in contact with peers via the Internet.” Respondents could answer on a 5-point scale that ranged from “should not” (1) to “should” (5).

*Perceived behavioral control* was measured directly with two items: “I consider myself capable of having contact with peers face-to-face” and “I consider myself capable of having contact with peers via the Internet.” Response options ranged from “totally disagree” (1) to “totally agree” (5). Perceived behavioral control was also measured indirectly by assessing *barriers*. In total, 13 items were formulated (see table 5). Respondents could answer on a 5-point scale that ranged from “very easy” (1) to “very difficult” (5). The barriers we asked about for face-to-face and online peer support partially differed, as a result of different characteristics. *Barriers to face-to-face peer support* was measured with 5 items (alpha = .83). *Barriers to online peer support* was measured with 8 items (alpha = .90).

### Data Analysis

Differences in Theory of Planned Behavior variables concerning face-to-face and online peer support were tested by means of paired-sample *t* tests. We used a hierarchical multiple regression analysis to determine to what extent intention to engage in peer support could be predicted. The determinants of the Theory of Planned Behavior were entered in the first block of the regression analysis. In the second block social support factors, health-related characteristics, and the demographic characteristics that correlated significantly with intention were entered. Statistical significance was assumed when *P* < .05.

## Results

### Participants’ Demographic and Health Characteristics

Most of the respondents were female (84.3%) (Table 1). The mean age of the respondents was 54 years. The majority of the respondents were married or living with a partner, had a low level of education, and were unemployed. Patients had a diagnosis of rheumatoid arthritis (40.1%), fibromyalgia (31.2%), or breast cancer (28.7%). The mean duration of the participants’ illness was 7 years, with a range from 0 to 59 years.

 The respondents had an average score of 38.6 on the physical component and an average score of 43.9 on the mental component of the SF-12v2. This indicates that the respondents’ physical and mental well-being was worse than the average of the general population.

**Table 1:** Demographic and health characteristics of the participants and social support factors (602 ≤ n ≤ 679)

**Sex, n (%)**		
	Female	571 (84.3%)
	Male	106 (15.7%)
**Age (years)**		
	Mean (SD)	54 (12.9)
	Minimum	18
	Maximum	75
**Marital status, n (%)**		
	Single	128 (19.5%)
	Married/cohabiting	530 (80.5%)
**Education, n (%)**		
	Low	404 (59.9%)
	Middle	176 (26.1%)
	High	94 (13.9%)
**Employment, n (%)**		
	Employed	212 (32.2%)
	Unemployed	447 (67.8%)
**Diagnosis, n (%)**		
	Breast cancer	195 (28.7%)
	Fibromyalgia	212 (31.2%)
	Rheumatoid arthritis	272 (40.1%)
**Disease duration (years)**		
	Mean (SD)	7.1 (7.8)
	Minimum	0
	Maximum	59
**Well-being (SF-12v2), mean (SD)**		
	Physical well-being	38.6 (11.3)
	Mental well-being	43.9 (6.7)
	Social support (score 1–4)	2.6 (0.66)

### Use of Face-to Face and Online Peer Support

The majority of the respondents (n = 396, 58.3%) had contact with peers during the past year (data not in table). The most regular type of peer support was contact with an acquainted peer (353/679, 52.0%) (Table 2). In total, 9.6% (65/679) of the respondents had engaged in face-to-face meetings for patients in the past year. Only 4.4% (30/679) of the respondents had contact with peers via the Internet in the past year. Of the respondents, 5.3% (36/679) indicated they had contact in the past year with peers at patient organization venues.

**Table 2:** Use of peer support (n = 679) during the preceding year

Type of peer support	Never		Once		Several times		Regularly	
	n	%	n	%	n	%	n	%
How often did you have contact with peers at patient meetings during the past year?	614	90.4	26	3.8	26	3.8	13	1.9
How often did you have contact with peers via the Internet during the past year?	649	95.6	7	1.0	17	2.5	6	0.9
How often did you have contact with (an) acquaintance(s) with the same disease during the past year?	326	48.0	35	5.2	216	31.8	102	15.0
How often did you have contact with peers at patient organization venues during the past year?	643	94.7	15	2.2	12	1.8	9	1.3

### Determinants of Theory of Planned Behavior Concerning Face-to-Face and Online Peer Support

The respondents’ intention to engage in face-to-face and online peer support in the coming year was slightly negative (Table 3). Only a minority of the respondents intended to look for peers via the Internet (35/654, 5.4% [certainly] yes; 135/654, 20.6% maybe; 484/654, 74.0% [certainly] not) or for face-to-face peer contact (104/663, 15.7% [certainly] yes; 164/663, 24.7% maybe; 395/663, 59.6% [certainly] not) in the coming year (data not in table).

**Table 3:** Mean scores (range 1–5) for determinants of the theory of planned behavior toward face-to-face and online peer support

Determinant	Face-to-face peer support (601 ≤ n ≤ 663)		Online peer support (530 ≤ n ≤ 654)	
	Mean	SD	Mean	SD
Intention^a^	2.4	1.2	2.0	0.95
Attitude^a^	3.7	1.1	3.2	1.1
Advantages^a^	3.5	0.85	3.2	0.86
Disadvantages^a^	2.9	0.88	3.2	0.85
Subjective norm^a^	3.0	0.69	2.8	0.67
Perceived behavioral control^a^	4.1	1.2	3.9	1.4
Barriers^b^	2.8	0.80	2.8	0.91

^a^ *P* < .001 for paired-sample *t* tests comparing face-to-face versus online peer support.

^b^ No differences in amount of barriers between face-to-face and online peer support could be determined, because the questionnaire asked about different barriers.

 Although the respondents had a slightly positive attitude toward both kinds of peer support, they were significantly (*P* < .001) more positive toward face-to-face support. Respondents experienced significantly greater advantages and fewer disadvantages using face-to-face support than using online peer support.

 The scores on the separate items (Table 4) revealed that the most important advantages of both types of peer support were “sharing experiences” and “finding recognition.”

**Table 4:** Mean item scores (range 1–5) on attitude toward peer support

		Face-to-face peer support (601 ≤ n ≤ 616)		Online peer support (526 ≤ n ≤ 546)	
		Mean	SD	Mean	SD
**Advantages: (Through) contact with peers...**					
	Offers a good opportunity to share your experiences	3.8	1.1	3.6	1.1
	provides recognition and understanding	3.8	1.1	3.6	1.0
	provides support	3.7	1.1	3.4	1.0
	is informative	3.5	1.1	3.4	1.0
	is comforting	3.4	1.0	3.1	1.0
	you feel empowered as a patient	3.4	1.1	3.1	1.1
	provides reliable information	3.3	1.1	3.1	1.0
	you can cope better with your illness	3.2	1.1	3.0	1.1
	you can accept your illness more easily	3.2	1.1	2.9	1.1
**Disadvantages:(Through) contact with peers...**					
	you are occupied too much with your illness	3.2	1.4	3.6	1.3
	is too informal	2.9	1.1	3.2	1.0
	makes people more concerned about the consequences of their disease	2.9	1.2	3.1	1.2
	is too shallow	2.7	1.1	3.2	1.0
	takes too much time	2.7	1.3	2.9	1.3

 The most important disadvantage of both types of peer support was the continual confrontation with their illness. In general, respondents felt significantly more encouraged by people in their social environment to be in contact with peers face-to-face than via the Internet. The respondents considered themselves significantly more capable of having contact with peers face-to-face than via the Internet.

 Differences in perceived barriers between face-to-face and online peer support could not be determined, because different barriers were asked about. The scores on the various barriers (Table 5) revealed that for both kinds of peer support, the most important one was to find a suitable peer support group. For online peer support other important barriers were to discuss the illness on the Internet, to actually write about the illness on the Internet, and the difficulty (due to the illness) of having to type or sit behind the computer for a long period of time.

**Table 5:** Mean item scores (range 1–5) for barriers to peer support

How difficult or how easy is it for you...		Mean	SD
**Face-to-face peer support (582 ≤n ≤595)**			
	to find a suitable face-to-face peer support group?	3.0	1.0
	to find the time and the opportunity to contact peers face-to-face?	2.9	0.97
	considering your illness to visit face-to-face peer support groups?	2.9	1.0
	to afford the cost involved with face-to-face peer contact?	2.8	1.0
	to talk about your illness with peers face-to-face?	2.7	1.0
**Online peer support (506 ≤n ≤518)**			
	to find a suitable online peer support group?	3.1	1.1
	to talk about your illness on the Internet?	3.1	1.1
	to verbally express your illness on the Internet?	3.1	1.1
	considering your illness to type or sit behind the computer for a long period of time?	3.1	1.2
	to find the time and the opportunity to contact peers via the Internet?	3.0	1.0
	to afford the costs involved with peer-to-peer contact via the Internet?	2.6	1.1
	to work with the Internet?	2.3	1.3
	to obtain access to the Internet?	2.2	1.2

### Prediction of Intention

Theory of Planned Behavior variables explained 33.3% of the intention to engage in face-to-face contact. Of the distal factors, physical and mental well-being, sex, and past behavior significantly improved the total amount of explained variance of intention to engage in face-to-face support. After inclusion of these distal factors, the influence of TBP variables remained significant (Table 6).

 Theory of Planned Behavior variables explained 26.3% of the intention to engage in online contact. Of the distal factors, mental health, age, and past behavior significantly improved the total amount of explained variance of intention to engage in online peer support. The influence of Theory of Planned Behavior variables on intention remained significant after inclusion of the distal factors.

 The total amounts of explained variance were moderate for face-to-face contact (40.0%) and online contact (36.2%).

 When repeating the analysis among only those patients who had not had (online) contact with fellow patients in the past, we found similar results: Theory of Planned Behavior variables explained 27.5% of intentions to engage in face-to-face support and 24.6% of online peer support (data not shown).

**Table 6:** Extent to which intention to seek online peer support and face-to-face peer support can be predicted by determinants of the theory of planned behavior

Determinant		Intention to seek face-to-face peer support (n = 554)			Intention to seek online peer support (n = 489)		
		*r*	Beta	SE	*r*	Beta	SE
**Step 1**							
	Attitude		0.22***	0.05		0.19***	0.05
	Advantages		0.17**	0.05		0.20***	0.05
	Disadvantages		–0.19***	0.04		–0.11**	0.04
	Subjective norm		0.22***	0.04		0.19***	0.04
	Perceived behavioral control		0.03	0.04		0.16**	0.05
	Barriers		–0.02	0.04		0.12*	0.05
		*R^2^* = .33, *F*_6,548_ = 45.1***			*R^2^* = .26, *F*_6,483_ = 28.7***		
**Step 2**							
	Attitude	.46**	.19***	0.05	.38**	.15**	0.05
	Advantages	.46**	.17**	0.05	.40**	.18***	0.05
	Disadvantages	–.33**	–.19***	0.04	–.19**	–.10*	0.04
	Subjective norm	.37**	.19***	0.04	.30**	.17***	0.04
	Perceived behavioral control	.19**	.00	0.03	.24**	.11*	0.04
	Barriers	–.18**	–.09*	0.04	–.07	.11*	0.05
	Social support factors	.02	–.06	0.04	–.02	–.01	0.04
	Physical health	–.14**	–.13**	0.04	–.23**	–.08	0.04
	Mental health	–.10**	–.09*	0.04	–.23**	–.12**	0.04
	Time since diagnosis	–.06	.07	0.04	–.14*	–.02	0.04
	Arthritis versus breast cancer	–.02	–.08	0.05	–.21**	–.05	0.05
	Arthritis versus fibromyalgia	.13**	.01	0.05	.30**	.03	0.06
	Sex (male vs female)	.11**	.08*	0.04	.03	–.04	0.04
	Age (years)	–.15**	.00	0.05	–.34**	–.14**	0.05
	Marital status (married/cohabiting vs single)	.07	.02	0.04	.09*	.00	0.04
	Support group past behavior	.30**	.15***	0.04	.27**	.13**	0.04
		*R^2^* = .40, *F*_16,538_ = 22.1***			*R^2^* = .36, *F*_16,473_ = 16.8***		
	*R^2^* change	.07***			.10***		

* *P* < .05, ** *P* < .01, *** *P* < .001.

## Discussion

To the best of our knowledge, this study is the first to examine which psychological determinants predict patients’ intention to engage in face-to-face and online peer support. Earlier studies focused only on determinants of patients’ intention to engage in face-to-face peer support, and frequently lacked a theoretical framework. Our study confirmed that only a relatively small percentage of the patients engaged in organized forms of peer support. The respondents were more positive about and more inclined to use face-to-face peer support than online peer support.

 Our results are in contrast to our expectations, as we had expected that the Internet and Health 2.0 technology would significantly facilitate peer contact between patients. In the literature, many advantages of online support groups are mentioned, such as easy accessibility, no physical or geographic barriers, and 24-hour availability. An explanation might be found in the fact that we questioned a somewhat older patient population. Older people mostly treat the Internet with greater skepticism than do younger people. “Trust” is of specific importance to patient support groups, because the topic of “an illness” in itself requires a high level thereof, thus this might have influenced patients’ perceptions of online peer support [25]. Another explanation may be that we only included patients with common diseases. For people who have a relatively rare disease, online peer support can provide a particularly valuable alternative, because for them it is more difficult to find peers with the same or similar conditions with whom they can share their experiences near their local communities [26].

 Our study revealed that in accordance with the Theory of Planned Behavior, having a more positive attitude, feeling more supported by people in the social environment, and feeling more able to participate in peer support increased the intention to participate in organized forms of peer support. However, it should be notified that perceived behavioral control is not significant for face-to-face support. This is in line with Grande et al [9], who found that a more positive attitude and a higher subjective norm increased engagement in (face-to-face) peer support. It is also in line with Voerman et al [10], who found that a more positive attitude and a higher perceived control increased intention to engage in peer support.

 Intention to engage in peer support was only modestly predicted by the Theory of Planned Behavior variables (face-to-face: 33.3%; online 26.3%). A meta-analysis has shown that Theory of Planned Behavior variables, on average, account for 35%–50% of the variance in intention [27]. It is difficult to compare the amount of explained variance with results of others studying participation behavior in face-to-face peer support, because in these studies logistic regression analysis was used [9,10]. An explanation for the relatively low amount of explained variance might be that, although respondents thought that peer support was valuable, they did not consider it valuable for themselves personally. According to the Theory of Planned Behavior, people need to perceive benefits of engagement in peer support to be of personal importance, instead of only for others, if they intend to execute the examined behavior [9,21]. In addition, future research might benefit from a combination of theoretical models to explain engagement in peer contact. In particular, the social comparison theory [28] has been used previously to study effects of peer contact, and could also be valuable in examining patients’ reasons for (not) participating in this type of contact. According to the social comparison theory, people have a drive to compare themselves with others who face similar challenges [28]. For patients this can lead to feeling “less alone” in coping with the disease [29]. In addition, upward social comparison (looking at people who are doing better) can be a source of inspiration and advice [30], while downward social comparison (looking at people who are doing worse) can lead to positive affect by providing examples of how bad things could be [31]. Although some of the assessed advantages and disadvantages in our measures did derive from social comparison theory, future studies could gain by more explicitly combining the two models.

 Patients who indicated having poorer mental well-being had a greater intention to participate in face-to-face and online peer support, and those who had worse physical well-being were more inclined to participate in face-to-face peer support. These results are not surprising, considering that health-related support groups have a health-promotional function. Therefore, these groups are less appealing to patients who perceive themselves already having good mental and physical well-being despite their illness [18,32].

 Of the demographic factors, only age significantly improved the total amount of explained variance of intention to engage in online peer support. Younger patients were more inclined to engage in online support groups. These results were in line with our expectations, as it is still mainly younger people who use the Internet [19].

### Pointers for an Intervention

This study yielded some pointers for an intervention, so that patients can make well-informed decisions about whether they want to engage in peer support and so that they can find a peer support group the moment they want to enroll. First, attention should be paid to awareness of peer support. Our study revealed that a considerable proportion of patients expected difficulties with finding relevant peer groups, especially on the Internet. Since studies showed that not all people have the necessary Internet skills to be capable of finding the information and applications they are looking for [33], it can be expected that not all patients manage to find online peer support groups by themselves. Second, our study revealed that many potential participants perceived various disadvantages to peer support. A major concern is the confrontation with negative sides of the disease. In line with Winefield et al [13], we believe that an intervention should inform potential participants of the specific aim of peer support groups and how they operate. Patients could, for example, be encouraged to read along with an online peer support group (ie, so-called lurking). By lurking, patients get a feeling for how such a group operates and what kind of people participate [34]. In addition, it could be emphasized that an increasing number of online peer support groups also offer the opportunity for “buddy matching.” An optimal peer match can have a positive influence on interpersonal trust, and this is an important basis for the exchange of experiences and empathy [35].

### Limitations

The findings of this study are limited by its cross-sectional nature. Therefore, we could attribute no causal relationships.

 A second limitation of this study is the high number of missing variables in the section of the questionnaire on TBP items concerning online peer support. A considerable number of people without computer skills did not respond to these items. In addition, findings for *disadvantages of online peer suppor* *t* might be less reliable because of the relatively low alpha (alpha = .65) of this construct.

### Conclusions

Although opportunities for having contact with fellow patients have been significantly increased by Health 2.0 Internet technology, only a minority of patients seem to be interested in organized forms of peer contact (either online or face-to-face). Patients seem somewhat more positive about face-to-face contact than about online contact.

 Our study revealed that in accordance with the Theory of Planned Behavior, having a more positive attitude and feeling more supported by people in the social environment increased the intention to participate in both kinds of peer support. In addition, perceived behavioral control influenced the intention to participate in online peer support. Nevertheless, we must conclude that the Theory of Planned Behavior variables only modestly predicted the intention to engage in face-to-face and online peer support.
